# Isolation, molecular detection and antimicrobial susceptibility profile of *Salmonella* from raw cow milk collected from dairy farms and households in southern Ethiopia

**DOI:** 10.1186/s12866-022-02504-2

**Published:** 2022-03-31

**Authors:** Alemayehu Gebeyehu, Mestawet Taye, Rahmeto Abebe

**Affiliations:** 1 Faculty of Veterinary Medicine, Hawassa University, Hawassa, Ethiopia; 2 School of Animal and Range Sciences, Hawassa University, Hawassa, Ethiopia

**Keywords:** Antimicrobial susceptibility, Culture, Ethiopia, *Inv*A gene and Salmonella

## Abstract

**Background:**

*Salmonella* is one of the foodborne pathogens affecting public health around the globe. A cross-sectional bacteriological study was conducted from December 2019 to November 2020. This study aimed to isolate, molecularly detect and determine antibiotic susceptibility patterns of *Salmonella* from raw cows’ milk collected from dairy farms and households in Hawassa, Arsi Negele, and Dale districts.

**Materials and methods:**

A total of 384 raw milk samples were collected using a simple random sampling technique. Standard bacteriological and biochemical tests were used to isolate *Salmonella*. The positive samples were further confirmed by the molecular test. Kirby-Bauer disk diffusion method was used for antimicrobial susceptibility testing of *Salmonella*.

**Results:**

Using bacteriological and biochemical detection tests, *Salmonella* was isolated from 10.42% (*N* = 40) of the total sample. However, in molecular detection, only 32 of the 40 isolates were confirmed to be *Salmonella* using PCR test. The prevalence was 8.54, 12.69, and 10.46% in Hawassa, Dale, and Arsi Negele districts, respectively. Bacteriological prevalence did not vary significantly between the districts (*P* > 0.05). Likewise, no significant (*P* > 0.05) variation was observed in the *Salmonella* isolation rate between households (12.5%) and farms (8.33%) as well as between dry (8.85%) and wet (11.98%) seasons. Based on herd size, the isolation rate of *Salmonella* was significantly higher (*P* < 0.05) in large-scale farms (19.51%) than in small (5.1%) or medium (5.6%) scale dairy farms. The result of the antibiotic susceptibility test showed that *Salmonella* isolates were 100% resistant to ampicillin, while they were 100% sensitive to ciprofloxacin. Multi-drug resistance (MDR) was demonstrated in all isolates.

**Conclusion:**

This study showed that *Salmonella* is widespread in the raw milk samples and developing MDR, which may be of public health concern in the study area. It is therefore important that dairy farmers and raw milk sellers in the study area take serious measures to avoid contamination of the milk with *Salmonella* spp. In addition, the active commitment of the animal health departments in the respective districts to sensitizing dairy farmers and the sensible use of antibiotics at the farm level can help to reduce the antibiotic resistance of *Salmonella* spp.

**Supplementary Information:**

The online version contains supplementary material available at 10.1186/s12866-022-02504-2.

## Background

Foodborne diseases are major public health problems in both developed and developing countries. More than 250 different foodborne diseases have been described. Most of these foodborne diseases are infectious diseases caused by a variety of bacteria [1]*.* Foodborne bacterial diseases are a critical problem to the public health [2]. Bacteria are commonly found in soil, water, plant, animals and animal products including milk, meat, cheese and yoghurt [1].

Cow milk has high water activity and nutritive value which serves as a kind medium for growth of microorganisms [3]. Microbes commonly isolated and detected from milk and milk products pose a critical problem to human health. Bacteria which are commonly isolated from milk includes *Escherichia coli, Staphylococcus aureus, Salmonella* spp. and *Listeria monocytogens* [4]. Among these pathogens *Salmonella* attributes, the major part of foodborne diseases [1].

*Salmonella* species belong to Gram negative, rod shaped, facultative intracellular bacteria that potentially infect a wide variety of hosts. *Salmonella* is comprised of two species, *Salmonella bongori* and *Salmonella enterica* [5]. Depending on the bacterial outer membrane somatic ‘O’ antigen, and flagellar ‘H’ antigen over 2700 different serovars of *Salmonella* has been characterized [6]. Out of these 2700 serovars, nearly 1500 belong to the *Salmonella enterica* subsp. Enterica [7]. *Salmonella enterica* subsp. enterica are the most common pathogenic and zoonotic bacteria causing different form of salmonellosis in human and animals [2].

Milk provided for human beings should not contain any pathogenic microorganisms [8]. However, raw milk and its products are considered as important sources of *Salmonella*. Milk and dairy products especially those produced from raw or unpasteurized milk have been attributed as potential vehicles for the transmission of *Salmonella* to humans [9, 10]. The contamination of raw milk by pathogenic microorganisms including *Salmonella* comes from feces of infected cattle, contaminated skin, infected udder, contaminated milking equipment, air, feed and water, and from milkers [11–13]. *Salmonella* is transmitted to human either through the fecal-oral route or through consumption of contaminated food (milk, eggs, and meats) and cause either typhoidal or nontyphoidal salmonellosis. In addition, milk is a potential source of multiple drug resistance, and is a potential public health concern. Antimicrobial resistance is one of the biggest global public health challenges [14].

Over the years, a number of studies have been carried out in Ethiopia that examine the occurrence of *Salmonella* in milk and feces of humans and cattle, as well as the development of patterns of resistance to various antibiotics in human and veterinary medicine [15–18]. However, the studies available are sparse given the serious public health threat posed by the organisms. In addition, data are not available for the city of Hawassa and its surrounding areas, which is one of the potential areas of the country for milk production and consumption associated with increasing urbanization. Further studies on *Salmonella* prevalence in milk and its antibiotic resistance profile are also believed to complement the baseline data in Ethiopia and worldwide. The aim of the present study was therefore to isolate and identify *Salmonella* from raw cow milk using cultural, biochemical and molecular methods and to determine the antimicrobial susceptibility pattern of the organism.

## Materials and methods

### Study area

For this study, Hawassa city and its neighboring districts (Dale and Arsi Negele) which supply milk for the big market at Hawassa were purposively selected due to their relatively larger potential for dairy cattle population and milk production. Hawassa is the capital city of the Sidama Region State, located at 275 km south of Addis Ababa. Geographically it lies between 7^0^3′1.35″ N latitude and 38^0^29′43.81″ E longitudes at an altitude of 1750 m above sea level. The annual rainfall and temperature vary from 800 to 1000 mm and 20.1–25 °C, respectively. Dale district is one of the potential milks producing districts in Sidama Regional State. The elevation of this district ranges from 1200 m above sea level along the shores of Lake Abaya to 3200 m at its westernmost point. Arsi Negele is a town in southeastern Ethiopia, located in the West Arsi Zone of the Oromia Region northern to Shashamane. This town is situated at a longitude of 38°42′E and latitude of 7°21′N and has an elevation of 2043 m above sea level. Arsi Negele town is the administrative center of Arsi Negele woreda [19].

### Source of milk samples

Raw milk samples for the present study were collected from households (households engaged in smallholder dairy farming primarily for household consumption) and dairy farms (specialized commercial dairy farms) found in Hawassa city, Dale and Arsi Negele Districts. For collection of milk from producers, appropriate number of dairy farms and households having one and more lactating cows were selected from the list of dairy farms and households in the study areas. The dairy farms and households were selected by using simple random sampling technique based on the data obtained from the district’s Livestock and Fishery Resource Development Offices. Prior to sample collection, a cooperation letter was sent to each district livestock and fishery resource development office. As a response, an animal health technician was assigned who helped during collection of milk samples from the dairy farms and households selected for the study.

### Study design and sampling method

General information about the total number of households, farms, farm size, and potential milk producing Kebeles was obtained from Livestock and Fishery Offices in the study area. Accordingly, in Hawassa city, three sub cities namely, (Hawella Tula, Tabor and Addis Ketema) were found to be the major milk producing sub cities. Likewise, Melka Sheki, Melka Giltota and Mali Weyo in Arsi Negele and Berra Tedicho, Wuhalimat, Mesencho, Manche and Shafina in Dale district were the other Kebeles identified for their relatively higher dairy potential. Therefore, these productive Kebeles were selected purposively for this study. Simple random sampling technique was employed to select farms and households in each Kebele except for large-scale farms where all such farms were included in the study. Similarly, milk containers were selected by simple random sampling to take appropriate raw milk samples. The whole study was carried out from December 2019 to November 2020. However, milk samples were collected during the dry (December, January, and February) and wet (July, August, September) seasons. The season in our study area is divided into two main seasons, wet and dry seasons, based on the average rainfall and average temperature. Here the dry season is a season of the year characterized by high temperature (28–29 °C) and low rainfall (24–44 mm), while the wet season is characterized by low average temperatures (24–25 °C) and high rainfall (128–140 mm). Accordingly, December, January and February are considered the dry season and July, August, September as the wet season [20]. For the purpose of this study and ease of data analysis, the farms were categorized as small scale (1–5 cows), medium scale (6–10 cows) and large scale (> 10 cows) [21].

### Sample size determination

The sample size required for this study was determined by considering a 50% expected prevalence of *Salmonella* in the study, 5% desired absolute precision and 95% confidence level using the formula given in [22] as follows.

$$\mathrm{n}=\frac{{\mathrm{Z}}^2\times {\mathrm{P}}_{\mathrm{exp}}\left(1-{\mathrm{P}}_{\mathrm{exp}}\right)}{d^2}$$

Where: n = required sample size; P_exp_ = expected prevalence; d = desired absolute precision,

z = statistic for a level of confidence =1.96.

The expected prevalence of *Salmonella* was considered 50% due to lack of similar previous study in the study area. Thus, the maximum number of raw milk samples needed to determine the prevalence of *Salmonella* was calculated to be 384 milk samples. Using proportional sampling method 164, 134 and 86 samples were allocated to Hawassa, Arsi Negele and Dale districts, respectively. Equal number of milk samples (*n* = 192) were collected from both households and farms. Likewise, equal number of milk samples (*n* = 192) were collected during dry and wet seasons.

### Milk sample collection and transportation

Utmost efforts were made to prevent contamination and cross contamination of milk in the course of sample collection. Samples were collected early in the morning around 7:00 to 8:00 AM by arranging time in communication with the milkers’ and owners of the farms. Nearly 10 ml of raw milk samples were collected into sterile screw capped bottles. The milk samples were then held in an icebox with ice packs and transported to Molecular Biotechnology Laboratory of School of Animal and Range Science, Hawassa University. All samples were clearly labeled with date of sampling, type of sample and with the name of household or farm. In the laboratory, raw milk samples were cultured immediately or stored at 4 °C for a maximum of 24 h until they were transferred into enrichment medium and inoculated onto a standard bacteriological media [23].

### Bacteriological isolation of *Salmonella*

Bacteriological examination was done according to microbiology of food chain; horizontal method for detection, enumeration and serotyping of *Salmonella* [23]. Accordingly, it is standard to use three stage processes: pre-enrichment, selective enrichment and selective plating to isolate *Salmonella*. In primary enrichment step; in order to get better recovery of *Salmonella* one ml of milk sample was measured aseptically, homogenized into 9 ml of buffered peptone water (HIMEDIA BM020, India) and incubated at 37 °C for 24 h. Likewise, in secondary enrichment step Rappaport-Vassiliadis with soya (RVS) was adjusted to room temperature according to the manufacturer’s directions. The mix incubated in the primary enrichment sample was well massaged by hand for at least 10 s. Then 0.1 ml aliquot was transferred and added it to 10 ml of Rappaport-Vassiliadis with soya (RVS). Finally, the tubes were vortexed and incubated at 41.5° for 24 h. Lastly, the enriched milk sample were plated onto a Selective Agar. Xylose lysine deoxycholate (XLD) agar (HIMEDIA M031, India) was used as selective medium for isolation of *Salmonella* and it was adjusted to room temperature according to the manufacturer. The secondary enrichment tubes were vortexed before plating on XLD agar. After adjusting XLD the samples were streaked from secondary enrichment tubes, using 10 μl loop and incubated at 35 °C for 24 h. After the recommended incubation time the selective-differential agar plates were examined for the presence of colonies meeting the description for suspect of *Salmonella* colonies. Typical *Salmonella* spp. colonies are pink colonies with or without black centers on XLD agar. Three to five typical colonies of *Salmonella* were picked and streaked onto Trypton soya agar and incubated at 37 °C for 18–24 h for the further biochemical identification.

### Biochemical identification

The biochemical identification was done according to [23, 24] by using indole, Methyl red, Vogas-Proskaur, urease, citrate utilization, triple sugar iron (TSI), lysine decarboxylase and hydrogen sulphide production tests.

### Antimicrobial susceptibility test

The antibiotic susceptibility tests of the *Salmonella* isolates were performed using Kibry-Bauer disk diffusion test on Muller Hinton agar (HIMEDIA M173, India). Pure colonies from trypton soya agar were taken with a wire loop, transferred to a tube containing 5 ml of saline water, and emulsified. The emulsified broth culture was incubated at 37 °C as far as it reached the 0.5 McFarland turbidity standard. Sterile cotton head swab was soaked into the emulsified broth and the bacteria were swabbed evenly over the surface of Muller Hinton agar plate within a sterile safety cabinet hood. The plates were put at room temperature for 15 min to allow drying. Antibiotic discs with known concentration of antimicrobials were carefully placed on the plates and the plates were incubated for 24 h at 37 °C. Each isolate of *Salmonella* was tested for a series of nine common antimicrobials. Chloroamphenicol (C) (25 μg), ampicillin (AP) (25 μg), cefotaxime (CTX) (5 μg), gentamycin (CN) (10 μg), streptomycin (S) (10 μg), kanamycin (K) (30 μg), nalidixic acid (NA) (30 μg), ciprofloxacin (CIP) (5 μg) and trimethoprim-sulphamethaxazole (TS) (25 μg), all from Oxoid company, United Kingdom. Following incubation, the diameters of clear zones produced by antimicrobial inhibition of bacterial growth were measured to the nearest millimeter for each disc using transparent straight-line ruler and then classified as resistant, intermediate, or susceptible according to published interpretive chart of clinical laboratory standard institute [25]. The MDR index was determined for each of the isolates examined using the formula: MDR index = x/y; Where “x” is the number of antibiotics to which the strain display resistance, and “y” is the total number of antibiotics to which the test strain had been evaluated for sensitivity [26].

### Molecular detection of *Salmonella*

*DNA extraction:* Genomic DNA extraction from *Salmonella* isolates were done by boiling and chilling as described by [27]. The DNA quality and concentration were detected using UV spectrophotometer (JENWAY Spectrophotometer, 6705).

*PCR amplification*: The primarily identified *Salmonella* was confirmed by PCR targeting *inv*A gene *of Salmonella* at genus level [28, 29]. The primer used for the amplification of the highly conserved region of *inv*A gene was Salm3 (forward): 5’GCTGCGCGCGAACGGCGAAG 3′ and Salm4 (reverse): 5’TCCCGGCAGAGTTCCCATT 3′ that produce an amplicon with expected length of 389 bp [28, 30]. A uniplex PCR condition was done to detect *invA* gene from *Salmonella* isolates as determined by [31] using a thermocycler (VWR UNO, 732–2549). Amplification was carried out in a total volume of 25 μl containing 0.7 μl of each primer (10 uM), 0.5 μl of dNTP mix (200 μM) with 1.5 mM MgCl_2_, 0.25 μl Taq DNA polymerase (0.05 U), 2.5 μl PCR buffer (1X), 5 μl template DNA and 20.25 ul of nuclease free water. A positive and negative control containing the template DNA from *Salmonella Typhimurium* ATCC 13311 (brought from Ethiopian Biodiversity Institute) and nuclease free water, respectively, was included in every experiment. The reaction condition was optimized with initial denaturation at 95 °C for 5 min followed by 35 cycles of denaturation at 95 °C for 1 min, annealing at 65 °C for 1 min and extension at 72 °C for 1 min. Finally, an additional extension was achieved for 7 min at 72 °C and stored at 4 °C infinitely.

*Electrophoresis of PCR products:* The PCR product (amplicons) was electrophoresed on a 1.5% agarose gel. After loading the amplicons and markers in each well, an electric current of 150 mA and 100 V was applied for 40 min. Electrophoresis results were observed under gel documentation system (GelDoc-It2 310 Imager, USA). The expected positive result was indicated by the presence of 389 bp band on the gel; 100 bp DNA ladder was used as a marker. The confirmed *Salmonella* isolate from each positive sample were stored at − 80 °C in 20% glycerol for further testing.

### Data management and analysis

The data generated from this study were entered and managed in Microsoft Excel Office 2016. All the data analysis was done using Statistical Package for Social Sciences (SPSS) software version 26. Descriptive statistics such as percentages and frequency distribution were used to describe the nature and the characteristics of data. The association of the *Salmonella* isolates with the source of milk samples (milk from dairy farms versus milk from households), season of sample collection, milk sample districts and farm size were analysed using Chi-square (χ^2^) test. In all the analyses, P - value less than 0.05 (*P* < 0.05) was considered as statistically significant.

## Results

### Overall isolation rate of *Salmonella*

A total of 384 raw milk samples were collected and bacteriologically examined to determine the isolation rate of *Salmonella*. Out of the total 384 raw milk samples examined, 164 (42.7%), 134 (34.9%) and 86 (22.4%) were collected from Hawassa, Dale and Arsi Negele, respectively. From the total of 384 raw milk samples examined, 40 (10.42%) were found to be positive for *Salmonella* by biochemical tests. Out of the 40 isolates, 14 (35.0%), 17(42.5%) and 9 (22.5%) were from raw milk samples collected from Hawassa, Dale and Arsi Negele districts, respectively. The isolation rate of *Salmonella* was relatively higher in raw milk samples collected from Dale district (12.69%) than from Arsi Negele (10.54%) and Hawassa (8.54%); however, the difference was not statistically significant (*P* = 0.51. Table 1).

**Table 1:** Overall isolation rate of *Salmonella* and its association with the district of raw milk samples

Milk sample district	Total milk sample examined	Number of positives	Isolation Rate (%)	χ^2^- Value	*P*-value
Hawassa	164	14	8.54	Ref.	
Dale	134	17	12.69	1.56	0.23
Arsi Negele	86	9	10.46	1.25	0.51
Total	**384**	**40**	**10.42**		

*Ref.* Reference, *χ*^*2*^ Chi-square.

### Association of *Salmonella* isolation rate with the origin of milk samples

Equal number of milk samples were collected from households (*n* = 192) and dairy farms (*n* = 192). Overall, the isolation rate of *Salmonella* was higher in raw milk samples collected from households (12.5%) than dairy farms (8.33%); however, the difference was not statistically significant (*P* = 0. 24) (Table 2).

**Table 2:** The association of *Salmonella* isolation rate with the source of raw milk samples

Source of samples	No examined	No positive	Prevalence (%)	χ^2^ -Value	P -value
Households	192	24	12.5	1.79	0.24
Dairy farms	192	16	8.33		
Total	**384**	**40**	**10.42**		

### Isolation rate of *Salmonella* in dry and wet seasons

It was observed that out of the total 192 raw milk samples collected during the dry season, 17 (8.85%) were found to be positives for *Salmonella*. Out of the positive samples, 6, 6 and 5 were from Hawassa, Dale and Arsi Negele districts, respectively. On the other hand, from the 192 raw milk samples collected during the wet season, 23 (11.98%) were found to be positive for *Salmonella*. Out of the 23 *Salmonella* isolates detected during the wet season 8, 11 and 4 were from Hawassa, Dale and Arsi Negele districts respectively. Though the overall isolation rate of *Salmonella* was higher during the wet season than the dry season, the difference was not statistically significant (*P* = 0.4) (Table 3).

**Table 3:** Association of the isolation rate of *Salmonella* with season of milk sample collection

Milk sampling season	No milk samples	No positive	Prevalence (%)	χ^2^	*P* Value
Dry	192	17	8.85		
Wet	192	23	11.98	1.0	0.4
Total	384	40	10.42		

### Isolation rate of *Salmonella* in relation to farm category

In this study, the isolation rate of *Salmonella* was determined in relation to farm categories. Based on herd size,79 small dairy farms, 72 medium scale dairy farms and 41 large-scale dairy farms were included in this study. The isolation rate of *Salmonella* in small, medium, and large-scale dairy farms was 5.06, 5.56 and 19.51%, respectively. The result showed that the isolation rate of *Salmonella* was significantly higher in large scale farms than that in small or medium scale dairy farms (*P* = 0.01) (Table 4).

**Table 4:** Isolation rate of *Salmonella* in relation to herd size of the farms

Herd size	No Examined	No positives	Prevalence (%)	χ^2^ -Value	P -value
Small	79	4	5.06	Ref.	
Medium	72	4	5.56	1.10	0.8
Large	41	8	19.51	4.45	0.01
Total	**192**	**16**	**8.33**		

*Ref.* Reference, *χ*^*2*^ Chi-square.

### Antimicrobial susceptibility test result

The antibiotic susceptibility tests of the *Salmonella* isolates were performed according to the Clinical and Laboratory Standards Institute guidelines by using Kibry-Bauer disk diffusion test on Muller Hinton agar. All the 40 *Salmonella* isolates were tested against nine commonly used antimicrobials. All the isolates were found resistant at least to one or more antimicrobials tested. The antibiotic susceptibility profiles of the isolates showed that the isolates were 100, 92.5 and 72.5% resistant to ampicillin, streptomycin and cefotaxime, respectively. On the other hand, all the isolates were 100 and 77.5% susceptible to ciprofloxacin and Trimethoprim-Sulphamethaxazole, respectively (Table 5).

**Table 5:** Antimicrobial susceptibility profile of *Salmonella* isolated from milk samples

Antibiotic tested	Status of antimicrobial agent against the isolates		
	Resistant (%)	Intermediate (%)	Susceptible (%)
Ampicillin	40(100)	-	-
Cefotaxime	31(77.5)	3(7.5)	6(15.0)
Chloroamphenicol	10(25.0)	17(42.5)	13(32.5)
Ciprofloxacin	-	-	40(100)
Gentamycin	17(42.5)	13(32.5)	10(25.0)
Kanamycin	26(65.0)	11(27.5)	3(7.5)
Nalidixic Acid	-	23(57.5)	17(42.5)
Streptomycin	37(92.5)	3(7.5)	-
Trimethoprim & Sulphamethaxazole	9(22.5)	0(0.0)	31(77.5)

Multiple antimicrobial resistances (resistance to two or more antimicrobials) were detected in 100% (40/40) of the isolates. Five different antimicrobial resistance patterns were observed (Table 6). From the total 40 isolates tested for antimicrobial sensitivity 3, 9, 8, 15 and 5 showed resistance to two, three, four, five and six antimicrobials, respectively. The highest multiple antibiotic resistance was seen in the pattern (AMP, CN, CTX, K, S) where nine isolates showed resistance to them. Furthermore, the MDR indexes of the isolates obtained from this study ranged from 0.22 to 0.66. MDR index values greater than 0.2 indicates unwise use of antibiotics.

**Table 6:** Multiple antimicrobial susceptibility profile of isolated *Salmonella*

Number of Antimicrobial Resistance	Antimicrobial Resistance Pattern (number of isolates)	Number of Isolates (%)
Two	AMP, CTX(2) AMP, S(1)	3(7.5)
Three	AMP, CTX, S(7) AMP, C,S(1) AMP, CTX, C(1)	9(22.5)
Four	AMP, CN, K,S(4) AMP, CTX, C, S(2) AMP, TS, K, S(2)	8(20.0)
Five	AMP,CTX,K,C,S(1) AMP,CN,CTX,K,S(9) AMP, TS, K, C, S(1) AMP, CTX, TS,K,S(4)	15(37.5)
Six	AMP,CN,CTX,K,C,S(3) AMP,CN,CTX,TS,K,S(1) AMP, CTX, TS,K, C, S(1)	5(12.5)

*Key to Abbreviations*: *AM*P Ampicillin, *C* Chloramphenicol, *CIP* Ciprofloxacin, *CN* Gentamycin, *CTX* Cefotaxime, *NA* Nalidixic Acid, *K* Kanamycin, *S* Streptomycin, *TS* Trimethoprim-Sulphamethoxazole.

### Polymerase chain reaction (PCR) confirmation test result

**Fig. 1:**
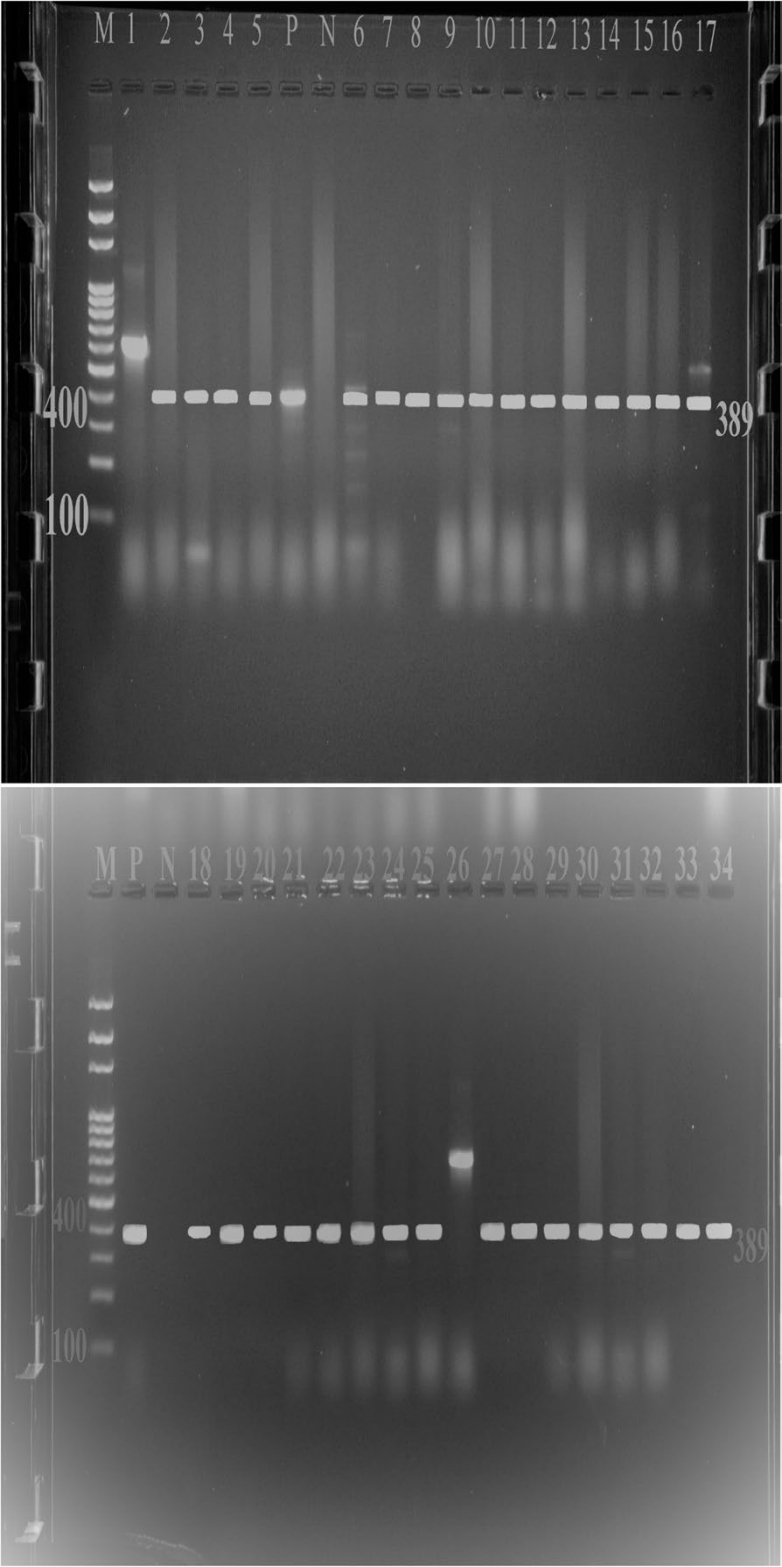
PCR amplification of biochemically identified *Salmonella*

Using the primers Salm3 and Salm4, a 389 sized PCR amplicon was obtained from DNA of *Salmonella* isolates (Fig. 1). PCR yielded a 389 bp fragment when DNA from *Salmonella* isolates was used as a template, whereas no specific products were obtained when other microbial DNA was used as a template. The *invA* gene was amplified from 32 out of 40 *Salmonella* isolates as shown in Fig. 1 below. Gels are cropped and put together from different tests which is included in supplementary information.

As shown in Fig. 1 above, lane 1 and lane 26 brought unexpected amplification with fragment length of 600 bp. However, all the other *Salmonella* isolates (Lane 2, 3, 4, 5, 6, 7, 8, 9, 10, 11, 12, 13, 14, 15, 16, 17, 18, 19, 20, 21, 22, 23, 24, 25, 27, 28, 29, 30, 31, 32, 33 & 34) and the positive control brought the expected amplicon length of 389. Therefore, 32 *Salmonella* isolates were confirmed by polymerase chain reaction.

## Discussions

Foods from animal origin are taken into account to be the major sources of foodborne salmonellosis. Therefore, routine detection of *Salmonella* in foods is an important part of public health programs. This cross-sectional bacteriological study was conducted to isolate and identify *Salmonella* from raw cows’ milk collected from selected dairy farms and households found in Hawassa city, Arsi Negele and Dale districts. The result of the current study shows that the overall isolation rate of *Salmonella* based on culture and biochemical tests was 10.42%, which is comparable to the findings of [17] who reported 10.5% in Modjo town. However, the prevalence of *Salmonella* in this study is relatively higher than the report of [16, 32, 33] who reported 6.0, 2.1 and 0.7% prevalence in Addis Ababa and Sebeta, respectively. On the other hand, reports from Iran (17.0%) by Hossein et al. (2013) and from Egypt (29.0%) by Omar et al. (2018) are much higher than the current investigation.

The difference in the relative occurrence of *Salmonella* in milk between the present and previous studies at different study areas in Ethiopia could be due to difference in the potential risk factors that contribute to the occurrence of *Salmonella*. For instance, milking procedure, milk handling practices, feeding strategies, hygienic and management practice, stocking density, usage of contaminated utensils, housing type, movement of animals, milking environment, and production facilities in different areas are the major risk factors that play a major role for *Salmonella* occurrence [18, 34]. Furthermore, the difference in the relative isolation rate of *Salmonella* may be due to the difference in the milk sample collected (since in the present study milk samples were taken from bulk milk whereas in the previous studies milk samples were taken from lactating cow), methods used, management and milk handling strategies in the study areas.

The study reveals that the milk sample sources is not significantly associated with the isolation rate of *Salmonella*. *Salmonella* is isolated regardless of milk sample source. The observed absence of variation might be due to similarity in hygienic and management practices, housing conditions, milking practices, feeding habits of the two farming systems. In commercial modern dairy farms, the hygienic and management practices are supposed to be better than the management practices in traditional farming system; however, in this study the isolation rate of *Salmonella* do not vary.

In this study, the isolation rate of *Salmonella* in raw milk samples were compared among milk sample districts (Hawassa, Arsi Negele and Dale). As a result, *Salmonella* is isolated irrespective of districts of milk sample collected without significant statistical association with *Salmonella* isolation. Here, the isolation rate of *Salmonella* from Arsi Negele, Dale and Hawassa is comparable. The possible explanation for the absence of variation among districts is supposed to be due to their comparable hygiene and management practices, housing conditions, milking and milk handling practices.

The association between the occurrence of *Salmonella* in milk and season of sample collection (dry and wet) were also determined in this study. Thus, the isolation rate of *Salmonella* is comparable in the dry and wet seasons with insignificant statistical difference. The ability of the bacteria to grow in a high range of temperature between 5 °C to 45 °C could be the possible reason why the prevalence does not vary between the two seasons.. Presumably, poor hygienic conditions on dairy farms and households have created an environment conducive for the organism to reproduce and persist. This, coupled with the ability of the bacteria to survive in wide temperature ranges, has led to contamination of the cows environment on the farms and thus of the milk regardless of the season. In contrast to the present findings, literatures show that contamination of bedding, feed and water containers, and gates and pens of the farm by the cattle dung is higher during the wet season [35].

In this study, we also further attempted to compare the isolation rate of *Salmonella* among farms with different herd size (small, medium and large). The isolation rate was comparable between small and medium sized farms. However, the isolation rate was significantly higher in large farms than in small or medium sized farms. This difference could be attributed to the difference in the bulkiness of milk, milk handling and management practices. Since samples were taken from bulk milk, the probability of cross contamination is considered to be high in large scale farms.

The increased score of antimicrobial resistance observed in *Salmonella* has become a public health concern. Antimicrobial utilization in animal production systems has long been suspected to be a cause of the emergence and spread of antimicrobial resistant *Salmonella*. Unwise use of antimicrobials in both human and veterinary medicine has contributed to development and dissemination of antimicrobial resistant pathogens [16, 36]. In this study, *Salmonella* isolates show high resistant to ampicillin, streptomycin and cefotaxime. The pattern of resistance to ampicillin observed in the current study is comparable to the reports of [16, 37]. However, the present resistance pattern to ampicillin is much higher than the findings of [17] who reported 39.5% in Modjo and that of [26] who reported 15.62% in India. This difference could be due to the differences in the habit of antimicrobials usage in animal production system in the study areas. Overall, *Salmonella* shows resistant to ampicillin and this is supposed to be due to long and extensive use in human and veterinary treatments over several years. In addition, the observed resistance of *Salmonella* to ampicillin may attribute to due to the acquired ability of the strains to produce β-lactamase enzymes that are able to degrade the chemical structure of the antimicrobial agents [38]. On the other hand, all the *Salmonella* isolates show highly sensitive to ciprofloxacin. This finding is in line with the reports of [16, 37] in which *Salmonella* isolates from human and cattle were 100% susceptible to ciprofloxacin. Though no data has indicated this, the effectiveness of ciprofloxacin may be because it is not widely used in countries like Ethiopia in the animal production system.

Over the years, bacterial pathogens have developed resistance against various antibiotics. In this study, all *Salmonella* isolates showed multiple drug resistance (MDR) to two or more antimicrobials. This is higher than the percentage of isolates showed MDR in previous studies in Ethiopia such as [16] (83.0%), [39] (36.4%), [40] (52.5%), [15] (31.8%), [17] (96.4%), and [37] (95.5%). This may be due to the increasing rate of inappropriate utilization of antimicrobials in the dairy animal production systems, which favor selection pressure that increased the advantage of maintaining strains of bacteria carrying resistance genes [41]. The MDR indexes of the isolates obtained from this study ranges from 0.22 to 0.66. MDR index values greater than 0.2 indicates unwise use of antibiotics [42]. Therefore, this study shows milk is a potential source of MDR, and is a potential public health concern in the study area.

The existence of *invA* gene in almost all *Salmonella* serovars and its absence from the other bacteria proved it as a genetic marker for the identification of *Salmonella.* In this study, the *invA* gene was amplified from 32 (80.0%) of the 40 *Salmonella* isolates. Eight isolates, which were biochemically, identified as *Salmonella* were excluded by polymerase chain reaction method. The possible explanation for this is; in the conventional bacterial isolation and biochemical characterization; *Salmonella* is confused with other related Enterobacteriaceae. However, PCR is highly sensitive because the *invA* gene is absent in other related bacteria. This amplification of *invA* gene using Salm3 and Salm4 primer is comparable with the reports of [28, 30].

The unexpected amplification in lane 1 and lane 26 is supposed to be due to attachment of the primer on non-target region which bring nonspecific product. Compared to conventional cultural and biochemical identification methods, PCR based method show better specificity, higher sensitivity, shorter analysis time, and better accuracy. Therefore, for detection of *Salmonella* in milk with better accuracy and specificity within short period of time polymerase chain reaction method is preferable.

## Conclusion

Detection and quantification of *Salmonella* in food samples should be regularly performed. The present study revealed that *Salmonella* are important contaminants of raw milk regardless of the source of milk, season of the year and districts sampled. However, the rate of isolation was significantly higher in large sized farms. The study further showed that the isolated *Salmonella* had developed varying degree of resistance to commonly used antimicrobials such as ampicillin, streptomycin, cefotaxime and gentamycin. It was marked that all of the isolates are resistant to two more antibiotics. Thus, the high percentage of MDR isolates recovered indicate the potential importance of raw milk as a source of MDR *Salmonella* infections and a serious public health concern and global challenge. It is therefore important that dairy farmers and raw milk sellers in the study area take serious measures to avoid contamination of the milk with *Salmonella* spp. In addition, the active commitment of the veterinary departments in the respective districts to sensitizing dairy farmers and the sensible use of antibiotics at farm level can help to reduce the antibiotic resistance of *Salmonella* spp. Further molecular studies are also needed to identify the *Salmonella* serotypes circulating in the study area.

## Supplementary Information

**Additional file 1**

## Data Availability

The datasets used and/or analyzed during the current study are available from the corresponding author on reasonable request.

## Abbreviations

PCR: Polymerase Chain Reaction
MDR: Multi-drug Resistance
*inv*A: Invasive A gene
XLD: Xylose lysine Desoxycholate agar
