# Supplementary material for: Isolation, molecular detection and antimicrobial susceptibility profile of Salmonella from raw cow milk collected from dairy farms and households in southern Ethiopia
Source: BMC Microbiol. 2022 Mar 31;22:84. doi: 10.1186/s12866-022-02504-2 (PMC8969351; doi:10.1186/s12866-022-02504-2)
Supplement: Supplementary file 1 — Additional file 1 [file 12866_2022_2504_MOESM1_ESM.pdf]

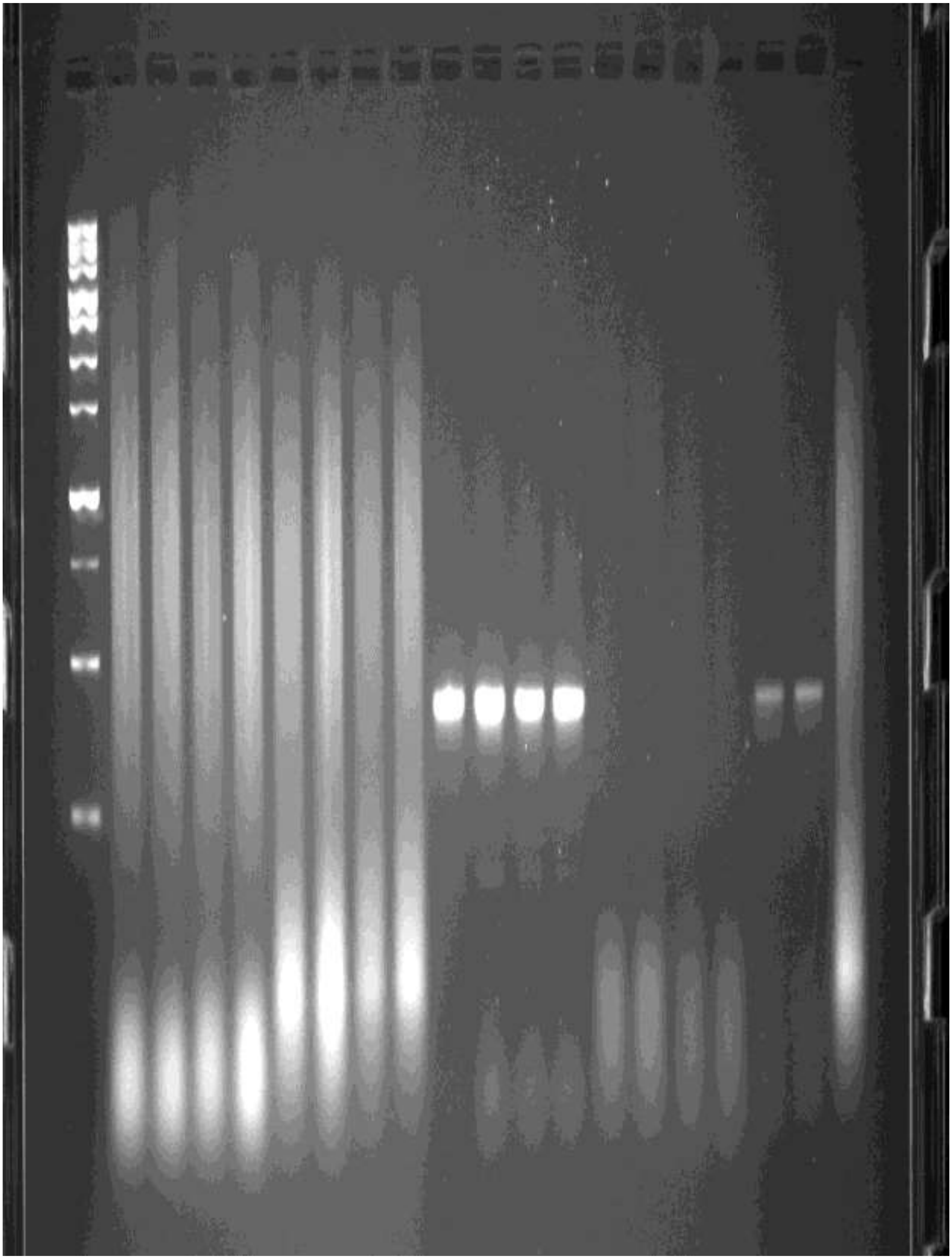

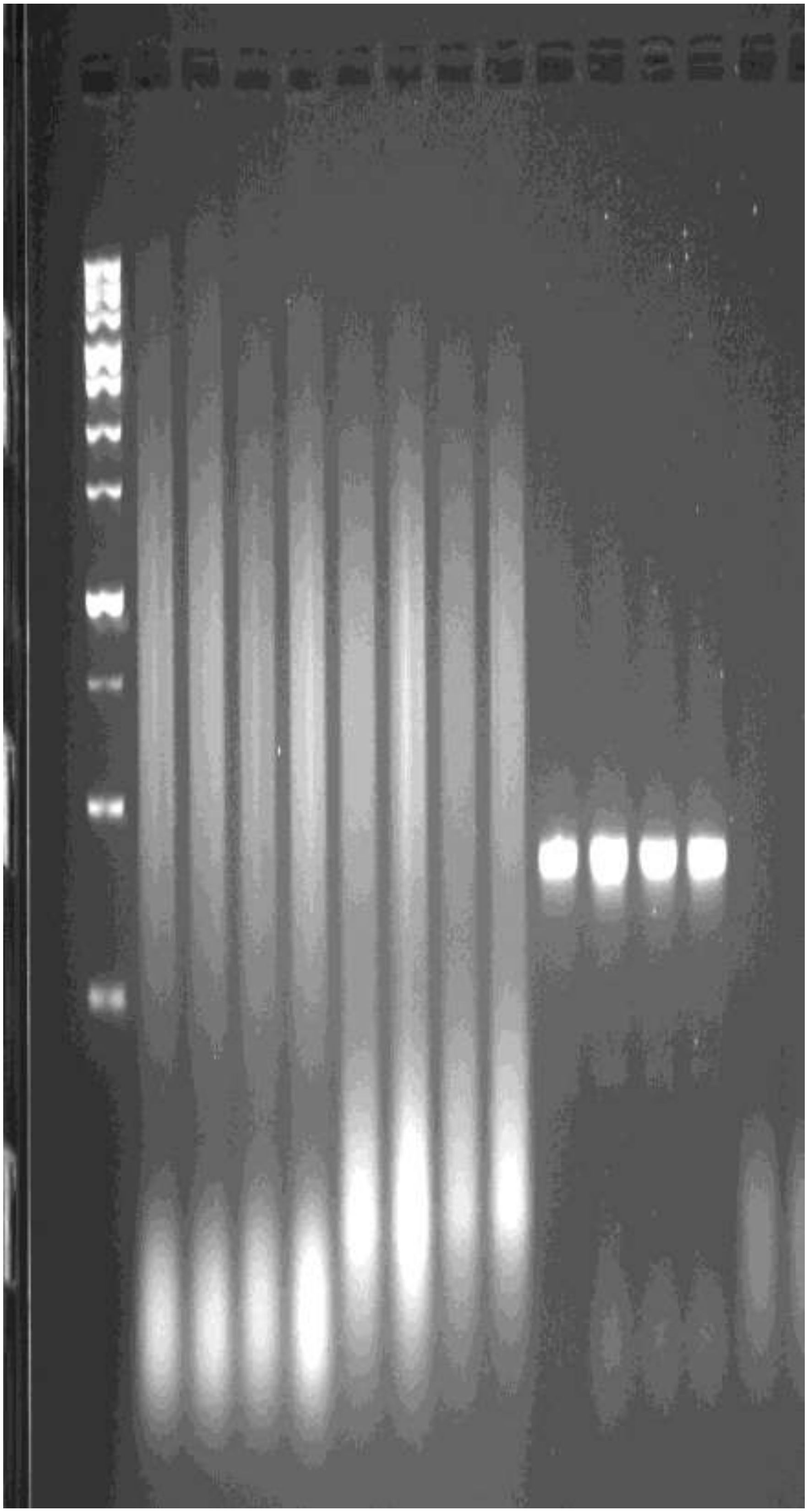

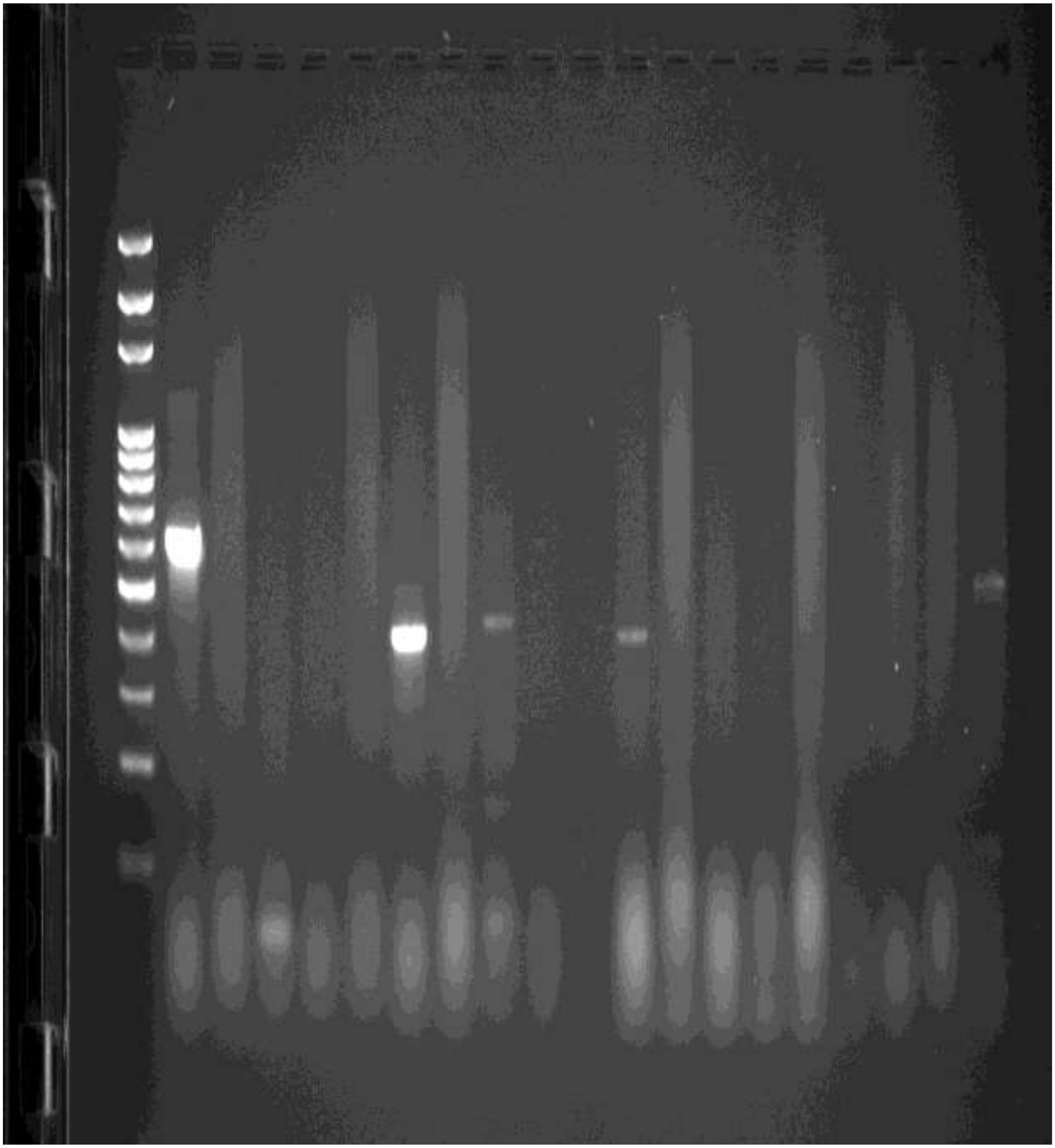

Figure 1: PCR amplification of biochemically identified *Salmonella*
Keys to abbreviations: M= Marker (100 bp ladder); P= Positive control; N= Negative control and lane 1-36= *Salmonella* isolates.
